# Combination Therapy, a Promising Approach to Enhance the Efficacy of Radionuclide and Targeted Radionuclide Therapy of Prostate and Breast Cancer

**DOI:** 10.3390/pharmaceutics13050674

**Published:** 2021-05-07

**Authors:** Tyrillshall S. T. Damiana, Simone U. Dalm

**Affiliations:** Department of Radiology & Nuclear Medicine, Erasmus Medical Center, Erasmus University, 3015 GD Rotterdam, The Netherlands; s.dalm@erasmusmc.nl

**Keywords:** combination therapy, targeted radionuclide therapy, peptide receptor radionuclide therapy, radionuclide therapy, prostate cancer, breast cancer

## Abstract

In recent years, radionuclide therapy (RT) and targeted radionuclide therapy (TRT) have gained great interest in cancer treatment. This is due to promising results obtained in both preclinical and clinical studies. However, a complete response is achieved in only a small percentage of patients that receive RT or TRT. As a consequence, there have been several strategies to improve RT and TRT outcomes including the combination of these treatments with other well-established anti-cancer therapies, for example, chemotherapy. Combinations of RT and TRT with other therapies with distinct mechanisms of action represent a promising strategy. As for prostate cancer and breast cancer, the two most prevalent cancer types worldwide, several combination-based therapies have been evaluated. In this review, we will provide an overview of the RT and TRT agents currently used or being investigated in combination with hormone therapy, chemotherapy, immunotherapy, and external beam radiation therapy for the treatment of prostate cancer and breast cancer.

## 1. Introduction

Radionuclide therapy (RT) involves the use of radionuclides that target specific cellular processes or accumulate in cancer cells because of their chemical properties [1]. Targeted radionuclide therapy (TRT) uses radiotracers that target molecules that are overexpressed on cancer cells. These radiotracers roughly consist of two main components, a targeting agent and a radionuclide [2]. The targeting agent can be, for example, an antibody, a natural or synthetic ligand, or a nanobody that can be coupled via a chelator to a radionuclide. There are different types of radionuclides used in medicine and biological research: those who emit α-particles (e.g., ^223^Ra, ^212^Bi, ^213^Bi, ^225^Ac, and ^227^Th), β^−^-particles (e.g., ^67^Cu, ^90^Y, ^131^I, ^177^Lu, ^186^Re, and ^188^Re), β^+^-particles (e.g., ^11^C, ^13^N, ^15^O, ^68^Ga, and ^18^F), auger electrons (e.g., ^111^In and ^125^I), and γ-emitters (e.g., ^67^Cu, ^177^Lu, and ^111^In) [3,4,5]. Radionuclides that emit β^+^-particles and γ-emitters are used in nuclear imaging. Besides, some of these radionuclides can emit both imaging photons and therapeutic particles (e.g., ^177^Lu and ^67^Cu). The selection of the optimal radionuclide for both imaging and treatment among other factors depends on the pharmacokinetic properties of the radiotracer. In addition, for treatment options, the type and stage of the disease are also important factors that need to be taken into consideration. In the treatment of large tumors, for example, β^−^-emitters are considered the ideal radionuclide. These β^−^-emitters have a long track length and therefore neighboring tumor cells that lack expression of the targeted molecules are also exposed to radiation, the so-called “cross-fire” effect [6,7]. For the treatment of micro-metastasis and small clusters of cancer cells, α-emitters, and auger electron-emitters are considered to be beneficial due to their short range and high-energy which results in a high level of cytotoxicity [8].

The broad option of targeting agents, together with the wide range of radionuclides with unique properties, allows radiotracers to be designed for targeting different types of cancers at different disease stages. Furthermore, a unique feature of RT and TRT is the ability to determine the radiation dose that reaches the tumor and healthy tissues, which can be assessed and correlated with efficacy and toxicity, respectively.

Besides having the most optimal radiopharmaceutical, for the treatment to be effective, several other factors should also be considered, including the cumulative radiation dose delivered, tissue and dose penetration, and the density of the targeted molecule on cancer cells. The delivery of radiopharmaceuticals might not always reach their target as desired due to heterogeneous expressions of the molecular marker that the radiotracer is designed to target and poor vasculature of the tumor [9]. Furthermore, studies have reported changes in radiosensitivity as well as a decrease in expression of the targeted molecule during disease progression and after exposure to other anti-cancer therapies [10,11,12,13,14,15,16]. The above-mentioned factors can greatly impact the success of RT and TRT, and as a consequence, novel strategies are needed to improve the treatment efficacy of RT and TRT. One promising strategy includes the combination of RT and TRT with other anti-cancer therapeutics that have shown promising results in tumor management [17]. This review will provide an overview of RT and TRT agents currently used or being investigated in combination with hormone therapy, chemotherapy, immunotherapy, and local external beam radiation therapy (EBRT) for the treatment of prostate cancer (PCa) and breast cancer (BC).

## 2. Prostate and Breast Cancer

PCa is the second most diagnosed cancer among men with an estimated 1,276,106 new cases and 358,989 deaths worldwide in 2018 [18]. The estimated PCa incidence and mortality rates per 100,000 males in 2018 were 29.3 and 7.6, respectively. For early detection of PCa, men at risk and between the age of 55–69 are recommended by their physician to be screened for PCa. The prostate-specific antigen (PSA) blood test is currently the first-line screening method used. Abnormal results of this screening are followed by prostate biopsy or multiparametric magnetic resonance imaging for further diagnosis [19,20]. Multiple treatment options exist for men diagnosed with PCa. These treatments are based on the stage and the grade of the tumor. Localized cancers are classified into three risk groups; low, medium, and high risk based on the Gleason score. Men with low-risk PCa are managed by “active surveillance” or “watchful waiting” while high-risk cancers receive more aggressive treatments such as surgery and radiation-based therapy. In advanced metastatic PCa, initial treatments include androgen deprivation therapy (ADT) and in some cases ADT in combination with chemotherapy. Besides these, several RT and TRT agents have been or are being investigated for the treatment of metastatic PCa [20,21,22]. A few RT agents have been accepted for clinical use, for example, ^223^Ra-dichloride and ^153^Sm-lexidronam for the treatment of metastatic castration-resistant PCa (mCRPCa) with bone metastases [23,24]. ^223^Ra-dichloride and ^153^Sm-lexidronam are both bone targeting agents that accumulate at sites with a high osteoblastic activity where they cause DNA damage in tumor cells [25,26]. Even though no TRT agents have been clinically approved for PCa treatment yet, preclinical and clinical studies show promising results. Among the TRT agents studied for PCa treatment, agents targeting the prostate-specific membrane antigen (PSMA) and the gastrin-releasing peptide receptor (GRPR) are the most well-known.

In females, breast cancer (BC) is the most diagnosed cancer and the leading cause of cancer death. There were an estimated 2,088,849 new cases and 626,679 deaths as a consequence of BC worldwide in 2018 [18]. For the early detection of BC, women at the age of 50 are recommended to be screened. Women at risk or who carry BRCA1 or BRCA2 mutations are advised to be screened as early as 40 years of age. The screening includes both physical breast examination as well as mammographic imaging. Abnormal results of these screenings are followed by biopsy for further diagnosis [27]. Both tumor morphologic characteristics and molecular factors are critical for treatment decisions in BC [28]. The disease is very heterogeneous and consists of multiple biological subtypes: luminal A, luminal B, human epidermal growth factor receptor 2 (HER2)-driven, and basal-like tumors [29,30,31,32]. Among other molecular factors, these subtypes can to some extent be identified by estrogen receptor (ER), progesterone receptor (PR), and HER2 expression, markers that majorly impact the treatment and prognosis of the disease. Luminal A tumors and luminal B tumors are either ER or PR positive, or both ER- and PR-positive. However, luminal A tumors either have higher ER- or PR-expression, or both, compared to luminal B tumors. Furthermore, luminal A tumors are HER2 negative, while luminal B tumors have variable HER2 expression. HER2-driven tumors are usually either ER- or PR-negative, or both ER- and PR-negative, and always HER2-positive. Basal-like tumors are the most aggressive subtype. These tumors are ER-, PR-, and HER2-negative, and are also known as triple-negative BC (TNBC). Tumors that are either ER- or PR-positive, or both ER- and PR-positive, are treated with endocrine therapy alone or in combination with chemotherapy [33]. Patients with either ER- or PR- positive, and HER2-positive tumors benefit more from a combination of hormone and anti-HER2 therapy, while TNBC tumors are treated with chemotherapy [32]. Unfortunately, treatment for metastatic BC remains a challenge. Currently, several markers including HER2, GRPR, and somatostatin receptors (SSTR) are being investigated as possible targets for TRT in the treatment of metastatic BC. Up to now, none of these TRT treatments have been approved for clinical use. Table 1 shows a selection of RT and TRT agents clinically used or preclinically/clinically studied for PCa and BC treatment.

## 3. Hormone Therapy-Based Combination Therapy

A key feature of PCa is its hormone responsiveness. The growth of PCa cells is usually dependent on androgens. This was observed first by Huggins and Hodges in 1941 [45]. Since then, ADT has become the primary treatment option for men with advanced metastatic PCa. Several strategies of ADT exist for the treatment of PCa. These strategies are classified into two groups, surgical castration, and medical/chemical castration. Surgical castration is achieved by orchiectomy, a surgical procedure in which one or both testicles are removed. However, its use is not so frequent and declining. Medical castration includes the use of anti-androgens (e.g., bicalutamide, flutamide), androgen synthesis inhibitors (e.g., abiraterone, enzalutamide), and luteinizing hormone-releasing hormone (LHRH) agonists and antagonists (e.g., leuprolide acetate, goserelin). Anti-androgens and drugs that target LHRH receptors represent first and second generations of ADT options. Androgen pathway inhibitors represent the third-generation of ADT drugs [46]. ADT is typically the first systemic treatment after local treatment options have become insufficient, although it is also used as neoadjuvant therapy for local radiation therapy and surgery [47,48,49].

Studies have shown that blockage of androgen can make tumors more radiosensitive. The radiosensitizing effect of ADT can occur through several mechanisms including cell cycle checkpoint inhibition, DNA damage repair pathway, and transcriptional changes in expression of DNA repair genes [50,51,52]. This provides a rationale for combining ADT with RT or TRT.

Once initiated, ADT is generally continued throughout the course of PCa treatment, including during metastatic PCa. Among other sites, bone is one of the most frequent metastatic target sites for PCa. It was reported that 90% of patients with metastatic PCa will develop bone metastasis [53]. The consequences are devastating, and the available treatment options are limited. Currently, ^223^Ra-dichloride is being used for the treatment of bone metastases [54]. However, patients continue to receive hormone therapy as well. A clinical study showed that ^223^Ra (50 kBq/kg) can be safely combined with abiraterone or enzalutamide for the treatment of bone metastasis. In this study, the addition of abiraterone or enzalutamide to ^223^Ra therapy demostrated prolonged median survival in patients with mCRPCa [55]. However, recently one study showed an increased incidence of fractures and deaths in patients who received ^223^Ra (55 kBq/kg) combined with abiraterone, compared with patients who received abiraterone alone [56]. The toxicity profiles of enzalutamide in combination with ^223^Ra are consistent with those seen when they are used as single agents. However, skeletal-related events of this combination are still under investigation (NCT02225704).

In a preclinical setting, LNCap cells treated in vitro with abiraterone or enzalutamide showed increased expression of PSMA which was linked to an antiproliferative effect. Interestingly, another cell line that is androgen-independent also increased its PSMA expression after abiraterone and enzalutamide treatment. However, in this case, no antiproliferative effect was observed [57]. Thus, patients especially those with advanced PCa who receive ADT may benefit from a combination of abiraterone or enzalutamide and PSMA-targeting radiotracers. In line with this Moo, T.A., et al., 2017 recently observed a seven-fold increase in ^68^Ga-PSMA-11 uptake in patients after receiving ADT [58]. Even though the PSMA radiotracer was used for imaging purposes in this study, these findings indicate the high potential of combination therapy when a PSMA radiotracer coupled to a therapeutic radionuclide is applied.

In BC, hormone therapy is given to patients whose tumors express ER, PR, or both. The purpose of hormone therapy is to prevent the interaction between estrogens and estrogen-dependent pathways that stimulate the proliferation of neoplastic cells. This can be performed by blocking the production of estrogens or by blocking the interaction between estrogen and cancer cells. The production of estrogen can be blocked by oophorectomy, aromatase inhibitors (e.g letrozole), or LHRH analogs (e.g., goserelin). The activity of estrogen towards tumor cells can be blocked using a selective estrogen receptor modulator (SERM) or a selective estrogen receptor down regulator (SERD) such as tamoxifen and fulvestrant, respectively [59,60].

Studies suggest that the cross-talk between estrogen and growth-factor signal cascades might inhibit the effects of radiation. Hence blockage of estrogen and thereby its cross-talk with growth-factor signal cascades can potentially make tumors more radiosensitive, providing reasoning for combining anti-estrogen treatment with RT or TRT [61].

Hormonal therapy is the first-line treatment for hormone-positive metastatic BC. As in PCa, in BC bone is also the most preferential metastatic site, and ^223^Ra in combination with hormone therapy is becoming more apparent in the treatments of bone metastasis in BC as well. A phase II study that evaluated the efficacy of ^223^Ra in combination with hormone therapy for the treatment of hormone-positive BC with bone metastases found a high disease control rate at nine months (49%) and tumor response rate at six months (54%) [62]. In this study, patients received ^223^Ra (55 kBq/kg) every four weeks up to six cycles and a hormonal agent, either tamoxifen or fulvestrant. In addition, these patients also received a subcutaneous injection of denosumab, a monoclonal antibody used for the treatment of osteoporosis, every four weeks, which was considered standard of care during the study period.

Interestingly, trastuzumab, an anti-HER2 in combination with ^177^Lu-DOTAGA-PEG_2_-RM26, a GRPR TRT, has been shown to prolong overall survival in mice bearing the human PCa PC3 xenograft [63]. This combination therapy strategy might also be interesting for targeting HER2 positive BC that are also ER or PR positive since it is known that this BC subtype often also expresses the GRPR [64,65,66].

## 4. Chemotherapy-Based Combination Therapy

**Figure 1:**
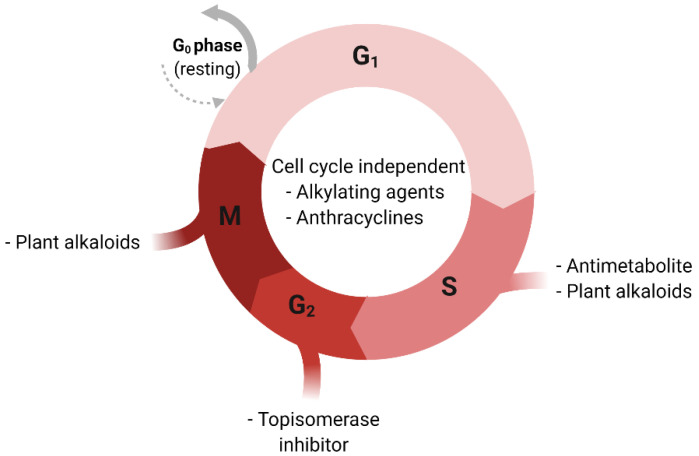
Cell-cycle and respective sensitivity to chemotherapeutic agents. G0; Gap 0 phase, G1: Gap 1 phase, G2: Gap 2 phase, S: Synthesis phase, M: Mitosis phase.

There are several types of chemotherapeutic agents used to treat cancer. These drugs can be categorized based on their chemical structure and their mechanism of action. Some of these drugs target different phases of the cell cycle while others are cell-cycle non-specific (Figure 1) [67]. Alkylating agents and platinum derivatives (e.g., cyclophosphamide and cisplatin, respectively) are active in the resting phase of the cell. These types of agents are therefore considered cell-cycle non-specific. Alkylating agents substitute alkyl groups for hydrogen atoms or bind to DNA causing crosslink formation within DNA double strands and thereby stop tumor growth. Platinum derivatives also cause crosslinking of DNA but do not alkylate [68]. Antimetabolites (e.g., 5-fluorouracil) block cellular metabolism by mimicking normal substances within the cell [69]. Anthracyclines (e.g., doxorubicin) act during multiple phases of the cell-cycle including during DNA replication [70]. Topoisomerase inhibitors (e.g., topotecan) are types of chemotherapy drugs that interfere with the action of topoisomerase enzymes (topoisomerase I and II). These enzymes are involved in the separation of DNA strands before DNA replication [71]. Plant alkaloids such as taxanes (e.g., paclitaxel and docetaxel) act during different phases of the cell cycle and are well-established cancer treatment options for both PCa and BC [71].

Understanding how chemotherapies work has helped scientists predict which drugs or therapies may be combined successfully. The combination of chemotherapy and radiotherapy has long been used in the treatment of several types of cancers [72,73,74,75,76]. The effect of this combination occurs through several mechanisms [67]. Chemotherapy and radiation can both induce DNA damage. Single strand breaks (SSBs) in the DNA caused by these therapies individually can normally be repaired rapidly. However, radiation-induced SSBs in the DNA can become more difficult to repair with the addition of chemotherapeutic agents such as alkylating agents and platinum derivatives, because DNA crosslinking caused by these chemotherapeutics hinder DNA damage repair. Furthermore, chemotherapeutic agents that effect nucleoside and nucleotide metabolism can inhibit post-radiation DNA damage repair. Moreover, agents such as taxanes exert their cytotoxic activity by preventing microtubule disassembly during the G2/M phase of the cell cycle, causing cell arrest. During these phases, cells are more radiosensitive because endogenous radioprotectors molecules are at their lowest. In addition, chemotherapies that target the S phase of the cell cycle and those that inhibit either proliferation, growth factor pathways, or both, may be effective in preventing tumor cell repopulation after radiotherapy, which is one of the crucial factors determining cure probability in radiotherapy [77,78]. Another benefit that chemotherapy brings when used in combination with radiotherapy is its ability to make hypoxic cells more radiosensitive. Tumor radiosensitivity rapidly decreases in hypoxic environments. The presence of oxygen can prevent repair of the DNA damage (oxygen makes the DNA damage permanent), the so-called “oxygen-enhancement effect”. This means that in hypoxic conditions, higher radiation doses are required to achieve the same biologic effect as in the presence of oxygen [79]. Chemotherapy can reoxygenate tumors by removing cells from the tumor allowing hypoxic cell to have more access to oxygen, and thus making them more sensitive to radiation [80,81].

As for RT and TRT, similar interactions to the above mentioned are expected. However, these might vary since the characteristics of RT and TRT (heterogeneous irradiation, long exposure, and low absorbed dose rate) differ from those of conventional radiotherapy (homogeneous irradiation, short exposure, and high absorbed dose rate) [82]. Nevertheless, there is growing evidence that supports the benefits of chemotherapy combined with RT or TRT. In an in vitro study, the combination of cisplatin and ^186^Re-HEDP (1.84 or 3.69 MBq/mL), a bone targeting agent, showed a synergetic effect in the treatment of R3327-MATLyLu cells [83]. These are metastatic anaplastic tumor (MAT) cells with high metastatic potential to the lymph nodes and lungs (LyLu) derived from the Dunning rat R3327 [84]. ^186^Re-HEDP and cisplatin alone both cause a decrease in the number of surviving tumor cells, and the combination of the two showed a greater effect in a dose dependent manner. The benefits of cisplatin in combination with bone targeting RT were later confirmed by a randomized clinical trial using ^89^Sr instead of ^186^Re-HEDP. They found that low-doses of cisplatin combined with ^89^Sr (148 MBq) significantly improved the quality of life and the duration of metastatic bone pain reduction in patients with CRPCa [85]. Pain relief was observed in 91% of the patients who received combination therapy and 63% in the placebo group with a median duration of 120 and 60 days, respectively. Furthermore, fewer new painful sites and less bone disease progression were observed when the combination therapy was administered.

Another study investigated the efficacy of docetaxel in combination with ^177^Lu-radiolabeled anti-Lewis Y (Le^Y^) human monoclonal antibody hu3S193 in mice bearing Le^Y^ positive DU145 PCa xenografts [86]. The Le^Y^ antigen is a blood group-related antigen that is overexpressed in over 60–90% of human epithelial cancers [87]. Treatment of DU145 xenografts with ^177^Lu-hu3S193 (3.7 MBq) alone caused a moderate delay in tumor growth of about 80 days. However, mice treated with the combination of ^177^Lu-hu3S193 and docetaxel survived until the end of the study and had a tumor growth delay of about 140 days. The authors also investigated the effect of the epidermal growth factor receptor (EGFR) tyrosine kinase inhibitor AG1478 in combination with ^177^Lu-hu3S193. EGFR is involved in regulating cellular proliferation, differentiation, and survival, thus blockage of the EGFR is expected to cause inhibition of cell growth [88]. This combination has shown to reduce the rate of tumor growth, however, to a lower extent compared to ^177^Lu-hu3S193 plus docetaxel. Furthermore, in other studies, docetaxel has shown to have a radiosensitizing effect in vitro in human colon, lung, head, neck, cervical, and several PCa cell lines [89,90,91,92,93].

In BC, chemotherapy is generally recommended as the first-line treatment for patients with TNBC. Patient-related factors such as tumor biology, disease growth rate, presence of metastases, menopausal status, and comorbidities should also be considered in the decision on a specific chemotherapy [94]. Anthracyclines alone, or in combination with other agents, are among the most active chemotherapies for the treatment of BC. One study investigated the effect of a monoclonal antibody targeting the EGFR, ^177^Lu-anti-EGFR in combination with docetaxel, doxorubicin plus the poly(adenosine diphosphate ribose) polymerase (PARP) inhibitor rucaparib (PARP inhibitors work by inhibiting DNA repair), for the treatment of TNBC cells [95]. ^177^Lu-anti-EGFR (300 MBq/kg) in combination with docetaxel and doxorubicin reduced the growth rate of MDA-MB-231 and HCI-002 BC xenografts by 83 days vs. 52 days when the BC xenografts were treated with ^177^Lu-anti-EGFR alone. The triple-agent combination therapy resulted in full eradication of the established xenograft. Furthermore, no recurrences were observed up to 120 days for MDA-MB-231 xenografts and >195 days for HCI-002 xenografts after the triple-agent combination treatment. Further analysis suggested that inhibition of central DNA repair proteins was crucial to the efficacy of the triple-agent combination.

Recently, ^177^Lu-Bombesin-PLGA (paclitaxel) nanomedicine has been investigated as a potential combination therapy for BC [96]. Here the radionuclide ^177^Lu was coupled to the bombesin (BN) peptide which is known to have a high affinity for the GRPR. High expression of GRPR is seen in several types of cancers including BC [97]. Paclitaxel (PTX) and, ^177^Lu-Bombesin were both loaded onto the poly lactic-co-glycolic acid (PLGA) nanobody and studied on MDA-MB-BC cells. The chemotherapeutic effect of PTX and the radiation of ^177^Lu-BN (5 MBq) produced by the ^177^Lu-BN-PLGA(PTX) nanosystem had a synergistic effect on MDA-MB-231 cell viability compared to BN-PLGA(PTX) alone. Interestingly, internalization with regard to total uptake was higher for ^177^Lu-BN-PLGA(PTX) compared to ^177^Lu-BN alone.

Furthermore, there is an ongoing clinical trial investigating the effect of ^223^Ra-dichloride (55 kBq/kg) in HER2 negative, hormone-positive BC patients with bone metastases treated with exemestane and everolimus (NCT02258451). The results of this study have not been published yet.

## 5. Immunotherapy-Based Combination Therapy

In recent years, immunotherapy has become an important cancer treatment. Several types of immunotherapy for cancer are known including monoclonal antibodies (e.g., anti-cytotoxic T-lymphocyte-associated protein 4), checkpoint inhibitors (e.g., anti-programmed cell death protein 1 (anti-PD-1) and its ligand programmed death-ligand 1 (anti-PD-L1)), cytokines, T-cell therapy, and vaccines. Infiltration into the tumor microenvironment and successful activation of effector T lymphocytes are crucial for the success of immunotherapy. However, tumor cells have developed a variety of mechanisms to reduce anti-cancer immunity [98]. A strategy to overcome this is by combining immunotherapy with other types of therapies that make tumors more T-cell inflamed, especially in PCa and BC since the majority of these tumors are not inflamed [99,100]. Several studies have shown that depending on the type of radiotherapy, radiation can induce a tumor-specific immune response or reprogram the tumor microenvironment in a way that facilitates both immune cell recognition and immune-mediated killing [101,102,103,104]. Furthermore, radiation can also induce systemic antitumor effects in unirradiated tumors/metastases after local radiation, known as the abscopal effect [105,106,107]. The radiation-induced abscopal effect occurs through several mechanism which involves the release of a number of endogenous damage-associated molecular patterns (DAMPs) by apoptotic cells via a process termed “immunogenic cell death” (ICD). Subsequently, DAMPs triggers dendritic cells (DCs) resulting in an enhanced tumor antigen presentation, and stimulates cytotoxic T lymphocyte and the release of cytokines and chemokines which all facilitates immune response to cancer cells [107,108].

In an in vitro study, ^223^Ra-dichloride has been shown to enhance T cell-mediated killing of PCa (LNCap and PC3), BC (MDA-MB-231 and ZR75-1), and lung cancer (H1703 and H441) cells [109]. These cell lines were first exposed to 4 or 10 gray (Gy) of ^223^Ra, then co-cultured with CD8^+^ effector T cells specific for carcinoembryonic antigen (CEA, HLA-A2-restricted), mucin-1 (MUC-1, HLA-A2-restricted), and brachyury (HLA-A2/A24-restricted) epitopes. This resulted in a significant increase in T cell-mediated killing after both 4 and 10 Gy of radiation. Furthermore, exposure to ^223^Ra significantly increased the expression of calreticulin and MHC-I, both molecules that facilitate and support the initiation of anti-cancer immunity [109,110,111]. Several clinical studies have/are investigating the combination of immunotherapy with ^223^Ra for the treatment of PCa. These combinations are mainly focused on abrogating immunosuppression using immune checkpoint inhibitors. A phase-I randomized clinical trial evaluated the safety and tolerability of the PD-L1 checkpoint inhibitor atezolizumab when given in combination with ^223^Ra-dichloride (55 kBq/kg) in patients with mCRPCa (NCT02814669). Although this study is completed, no results have been published yet. In addition, a phase-II study is assessing the antigen-specific immune response of sipuleucel-T vaccine therapy with or without ^223^Ra (50 kBq/kg) (NCT02463799), whereas another phase-II study is investigating the combination of ^223^Ra plus the anti-PD-1 checkpoint inhibitor pembrolizumab as a possible treatment for castration-resistant PCa (NCT03093428).

In a preclinical study, the checkpoint inhibitor anti-PD-1 in combination with a PSMA TRT in mice bearing PCa tumor was investigated [112]. Anti-PD-1 in combination with ^225^Ac-PSMA-617 (30 kBq) demonstrated improved tumor control compared to the monotherapies. The time to tumor progression was 47.5 days in the combined therapy group vs. 33.5 days with anti-PD-1 or 30 days with ^225^Ac-PSMA-617 alone. In line with this, survival was extended to 51.5 days when animals received combination therapy vs. 37 and 32 days, respectively, when animals were treated with anti-PD-1 or ^225^Ac-PSMA-617 alone.

To our knowledge, there are no studies that have reported on the combination of immunotherapy with RT or TRT in BC. However, the findings gained from the studies with PCa could provide crucial insight for the development of these kinds of combination therapies for BC as well.

## 6. External Beam Radiation Therapy-Based Combination Therapy

Besides hormone therapy, chemotherapy, and immunotherapy, radiation therapy or radiotherapy remains an important therapeutic option for cancer treatment. About 50% of all cancer patients will receive radiotherapy of some kind throughout the period of their illness [113]. There are several types of EBRT including 3D conformal radiotherapy, intensity-modulated radiation therapy, image-guided radiotherapy, stereotactic body radiation therapy, photons radiation, and particle radiations [114]. EBRT is commonly used in everyday radiation therapy treatment. The biological effect of radiation, in general, is DNA damage. If the radiation-induced DNA damage is irreparable, the cell will eventually die during cell division. Thus, the higher the dose the higher is the chance of irreversible DNA double-strand breaks (DSBs). However, the applicable dose of EBRT is limited by the radiosensitivity of the surrounding healthy tissue. The addition of RT or TRT to EBRT offers the possibility to increase the dose applied to the solid tumor without exceeding the limitations of the surrounding normal tissues. Another advantage of this combination is that it can be directed against both localized tumors and metastases [115].

With regard to the literature, reports about EBRT in combination with RT or TRT for the treatment of PCa are rare. A phase one study investigated EBRT (8, 20, or 30 Gy) in combination with ^223^Ra (50 kBq/kg) in patients with hormone-refractory PCa needing EBRT due to bone pain [116]. EBRT in combination with ^223^Ra caused a significant reduction in bone-alkaline phosphatase concentration compared to EBRT alone. The median time to first skeletal-related events such as bone pain and bone fractures was 14 weeks in the combined treatment and 11 weeks for EBRT alone. Furthermore, overall survival was higher for patients who received EBRT in combination with ^223^Ra (65 weeks) compared to EBRT alone (46 weeks). Another group also observed a reduction in pain intensity in patients with mCRPCa after being treated with EBRT (8–30 Gy) in combination with another bone targeting radionuclide, ^153^Sm (37 MBq/kg) [117]. Toxicity profiles of this combination therapy were acceptable and fully reversible. Hematological toxicity analysis demonstrated no statistically significant differences between the combination therapy and the monotherapy in hemoglobin concentration and the number of blood cells. Other adverse events such as hypercalcemia were reported among a few candidates in both study arms, but no pathological fractures or spinal cord compression was observed in these patients.

As for BC, Cornelissen, B., et al., 2004 were able to increase the efficacy of EBRT by combining it with a TRT targeting the DNA damage response protein, γH2AX, using anti-γH2AX antibodies conjugated to ^111^In [118]. Here, MDA-MB-468 BC cells were exposed first to ^111^In-DTPA-anti-γH2AX (6 MBq/μg) after which EBRT (10 Gy) was applied. Immunostaining for γH2AX showed an increase in the number of γH2AX foci/cells in the combination therapy compared to EBRT alone, which is an indication of more DNA DSBs. This was confirmed with a clonogenic assay which showed a reduction in the survival rate of MDA-MB-468 BC cells. Furthermore, the combination EBRT plus ^111^In-DTPA-anti-γH2AX significantly decreased the growth rate of MDA-MB-231 xenografts in vivo compared to the single agents. However, this strategy may not be effective in metastatic disease, because only cells in the radiation field will have increased γH2AX after EBRT.

## 7. Discussion and Conclusions

The rising interest in TRT has led to the evaluation of several combination strategies. Besides the above-mentioned combination therapies for PCa and BC, other interesting combinations of TRT with other anti-cancer drugs have been studied for targeting of other cancer types. The knowledge gained from these studies can potentially be used to develop promising combination treatments for PCa and BC in the future. Well-known TRT agents that have been widely studied in combination are those that target the SSTR. SSTRs are G protein-coupled receptors with five known subtypes (SSTR1–5). They are found in several tissues including neuroendocrine tumors (NETs) and to a lesser extent in BC. Because of their high expression in NETs, several radiolabeled SST analogs have been developed over the years, with octreotate having the highest uptake. ^177^Lu-DOTA^0^-Tyr^3^-Octreotate also known as ^177^Lu-octreotate is the first TRT approved by the EMA and the FDA for the treatment of SSTR-positive gastroenteropancreatic NETs [119]. Even though ^177^Lu-octreotate has proven successful in the treatment of several NETs [120,121,122], only a limited number of patients show complete remission after the treatment [123,124]. Therefore, many approaches that combine SSTR TRT with other therapies are being explored to improve its therapeutic efficacy. Capecitabine (CAP) and temozolomide (TEM) in combination with ^177^Lu-octreotate have been investigated by several groups [125,126,127,128,129,130,131,132] with some showing tumor response rates that are higher compared to ^177^Lu-octreotate alone, the latter reported by Kwekkeboom, D.J., et al., 2008 [120]. A pre-clinical study evaluated what would be the most effective treatment schedule for TEM and SSTR TRT combination therapy [133]. In this study, treatment with TEM resulted in increased tumor perfusion which reached its peak after 14 days. At this time point, the uptake of ^177^Lu-octreotate was also at its peak in the combined therapy group. The peak of radioactivity uptake was not influenced by tumor volume since the average tumor size was in the same range at day 0 and day 13. An increased level of SSTR2 expression was not the reason for the increased uptake of ^177^Lu-octreotate as no difference in SSTR2 expression prior to vs. after TEM treatment was observed. However, the cell line (H69) used in this study already expresses high levels of SSTR2 which may not increase further after TEM treatment. In line with this, another group did show a positive correlation between SSTR2 expression and ^177^Lu-octreotate uptake in low SSTR2 expressing cells after TEM treatment [134].

Several studies have examined the influence of other chemotherapies on SSTR expression and uptake of radiotracers. However, the results of these studies are variable. One study showed that several pancreatic tumor cell lines reduced the expression of high-affinity binding sites for ^111^In-labeled lanreotide derivative DOTA-LAN, another radiolabeled SST analog, after being treated with gemcitabine, 5-fluorouracil, cisplatin, camptothecin, mitomycin C, and doxorubicin [135]. In a different study, upregulation of SSTR2 expression in three different NET cell lines was demonstrated after treatment with 5-fluorouracil, however, in combination with epigenetic modifiers such as decitabine or tacedinaline [136].

The above-mentioned chemotherapeutic agents investigated in combination with radiolabeled SST analogs are used to treat different types of cancers, including PCa and BC. To the best of our knowledge, no studies have investigated whether these specific chemotherapeutic agents can cause similar effects in regard to the upregulation of SSTR2 expression in BC. Furthermore, it would be very interesting to know what the effects of these agents are on other receptors that are expressed by PCa (e.g., PSMA and GRPR) and BC cells (e.g., GRPR). Tumor perfusion should also be taken into consideration since this can potentially lead to higher doses of radiopharmaceuticals at the tumor site, even when target expression levels remain the same.

Radiation can also increase SSTR expression. Two studies demonstrated up-regulation of SSTR1, SSTR2, and SSTR5 expressions on NET cells after being treated with ionizing radiation in vitro [137,138]. Thus, irradiation of tumor tissue could be used to increase the specific binding of subsequent administrated ^177^Lu-octreotate, resulting in an increased absorbed dose to the tumor cells. A few studies investigated whether pre-treatment or priming with ^177^Lu-octreotate could induce similar effects. One group demonstrated higher uptake of ^111^In-octreotate following an injection of a low non-curative amount of ^177^Lu-octreotate. Priming with a high ^177^Lu-octreotate concentration resulted in lower uptake of ^111^In-octreotate [139]. However, a follow-up study found no statistically significant difference in SSTR expression between treatment with and without priming [140]. This indicates that the increase in tumor uptake here might not be due to increased levels of SSTR but rather another mechanism. Nevertheless, a potential approach to increase the efficacy of TRT might involve treatment with EBRT followed by fractionated treatment with TRT.

As was also previously mentioned, radiation can facilitate anti-cancer immunity via a range of cellular mechanisms of both tumor and immune cells [101,102,103,104]. It has been demonstrated, that in contrast to high radiation doses, lower doses of radiation induce several changes within tumor cells that ultimately facilitate immune cell recognition and immune-mediated tumor killing [141,142]. These results are promising and offer a great opportunity for the use of RT or TRT in combination with immune therapy. However, studies showed that radiation can also lead to immunosuppression. It is therefore very important to take the immunosuppressive mechanisms of radiation into account when designing combination therapies. For example, radiation has been shown to activate the transforming growth factor β and promotes the accumulation of regulatory T cells and pro-tumorigenic macrophages [143,144,145]. Fortunately, there is a promising approach to overcome these immunosuppressive mechanisms of radiation. This approach involves the use of the before-mentioned immune checkpoint inhibitors such as the anti-PD-1 in combination with radiation. Clinical trials have shown safety and impressive anti-tumor responses of antibodies blocking the activity of PD-1 or PD-L1 [146,147], and others have shown increased levels of PD-L1 in tumors after local irradiation [148]. This provides a clear rationale for combining RT or TRT with immune checkpoint inhibitors, for example, as was done for the combination of ^225^Ac-PSMA-617 with anti-PD1 [112]. However, a considerable amount of work is still required to define the optimal dose and radionuclide that can create maximal interactions with the immune system and to identify the type and schedule of immunomodulatory drugs that are best suitable for combination treatment.

Besides, the development and application of RT and TRT itself face many challenges as well. For example, a short half-life of radiotracers in the blood circulation can be a major obstacle since this will lead to deterioration of radiotracers before reaching the intended target. The loss or altering of binding affinity to the target due to in vivo metabolism are other potential challenges. Specific uptake of radiotracers in healthy organs expressing the target can be dose-limiting and reduce effectiveness. Finally, small radiolabeled molecules such as peptides can cause renal toxicity due to uptake and retention of radionuclides in the kidneys as a consequence of renal clearance and partial reabsorption, while larger radiolabeled molecules such as antibodies can cause hematological toxicity because of their long circulation half-life

In conclusion, there is still a significant amount of work that needs to be done in order to fully benefit from combination therapies with RT and TRT for PCa and BC. Nevertheless, the knowledge gained from current studies can help identify promising combination therapy strategies that can be further explored to improve the efficacy of RT or TRT and thus clinical outcomes of PCa and BC in the future.

## Figures and Tables

**Table 1:** Selection of RT and TRT agents on the market or under development for the treatment of PCa and BC.

Agent	Type of Cancer	Target	Disease Stage	Development Phase	Trial Name or Registration Number	Ref.
Radionuclide therapy						
^223^Ra chloride	PCa	Calcium analog	Bone metastasis	Commercially available	-	[34]
	BC			Clinical study	-	[35]
^153^Sm-lexidronam	PCa/ BC	Binding to hydroxyapatite matrix	Bone metastasis	Commercially available	-	[36]
Targeted radionuclide therapy						
^177^Lu-PSMA-617	PCa	PSMA	mCRPCa	Phase III; active, not recruiting	NCT03511664	
^227^Th- PSMA-TTC	PCa	PSMA	mCRPCa	Phase I; recruiting	NCT03724747	
^177^Lu-PSMA/CTT1403	PCa	PSMA	mCRPCa	Phase I; active, not recruiting	NCT03822871	
^177^Lu-PSMA-R2	PCa	PSMA	mCRPCa	Phase I/II; recruiting	NCT03490838	
^177^Lu-J591/TLX591/ ^177^Lu-DOTA-Rosopatamab	PCa	PSMA	mCRPCa	Clinical study	-	[37]
^225^Ac-PSMA-617	PCa	PSMA	mCRPCa	Clinical study	-	[38,39]
^177^Lu-NeoB, formerly known as ^177^Lu-NeoBOMB1	PCa	GRPR	Under investigation	Phase I/II; Recruiting	NCT03872778	
^177^Lu-NeoB/NeoBOMB1	PCa, BC	GRPR	Under investigation	Preclinical study	-	[40,41]
^177^Lu-RM2	PCa	GRPR	mCRPCa	Clinical study	-	[42]
^177^Lu-DOTA^0^-Tyr^3^-Octreotate and ^177^Lu-DOTA-JR11	BC	SSTR	Under investigation	Preclinical study	-	[43]
^188^Re-trastuzumab	BC	HER2	Under investigation	Preclinical study	-	[44]

RT: radionuclide therapy, TRT: targeted radionuclide therapy, PCa: prostate cancer, BC: breast cancer, PSMA: prostate-specific membrane antigen, GRPR: gastrin releasing peptide receptor, SSTR: somatostatin receptor, HER2: human epidermal growth factor receptor 2, mCRPCa: metastatic castration-resistant prostate cancer.

## Data Availability

Not applicable.
